# Erythrodermie und Blasenbildung bei einem Neugeborenen

**DOI:** 10.1007/s00105-023-05141-6

**Published:** 2023-05-11

**Authors:** Irina Gasslitter, Benno Kohlmaier, Bernhard Schwaberger, Robert Gruber

**Affiliations:** 1 Universitätsklinik für Dermatologie, Venerologie und Allergologie, Anichstr. 35, 6020 Innsbruck, Österreich; 2 Klinische Abteilung für Neonatologie, Medizinische Universität Graz, Graz, Österreich

## Anamnese

Wir berichten von einem männlichen Frühgeborenen, das im September 2021 mit einem Gestationsalter von 35 + 4 Schwangerschaftswochen und einem Geburtsgewicht von 2650 g (20. Perzentile) geboren wurde. Schwangerschaftsverlauf und Geburt waren unauffällig. Die Eltern waren hautgesund und verneinten eine Konsanguinität.

## Klinischer Befund

**Figure 1:**
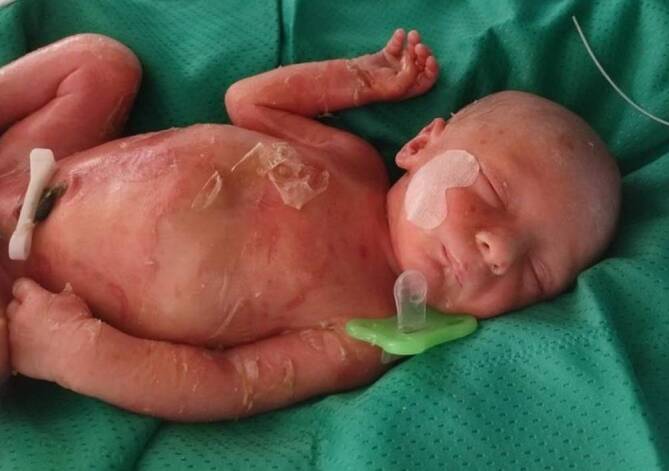

Klinisch zeigte sich eine ausgeprägte Erythrodermie mit generalisiert vorliegenden schlaffen, mehrere Zentimeter großen Blasen v. a. am Stamm und Erosionen betont an den Extremitäten (Abb. 1). Es zeigten sich keine Kollodiummembran, keine Ohren- und Augendeformitäten, kein Eklabium, kein Ektropium und keine palmoplantaren Hyperkeratosen. Weitere ektodermale Strukturen waren unauffällig.

## Diagnostik

Zur Diagnosesicherung wurden Abstriche, Blutkulturen sowie serologische und molekulargenetische Analysen durchgeführt. Da sich der Hautstatus im Inkubator innerhalb weniger Tage verbesserte und um eine Infektionsquelle zu vermeiden, wurde auf eine Hautbiopsie verzichtet. Alle Abstriche, Blutkulturen und serologischen Untersuchungen waren unauffällig.

## Verdachtsdiagnosen

Es wurde die Verdachtsdiagnose einer kongenitalen Ichthyose, Typ keratinopathische Ichthyose gestellt. Differenzialdiagnostisch musste an eine Epidermolysis bullosa, toxische epidermale Nekrolyse, ein „staphylococcal scaled skin syndrome“, weitere Infektionen mit Bakterien (Listerien, *Haemophilus influenzae*), Pilzen oder Viren (HSV (Herpes-simplex-Virus), VZV (Varizella-Zoster-Virus), Coxsackie, CMV (Zytomegalievirus)), eine (bullöse) Skabies oder eine Lues congenita gedacht werden.

## Weitere Befunde

Die Exomsequenzierung mit HPO-Term-Auswertung zeigte die heterozygote Missense-Mutation *c.562A* *>* *G *im* KRT1*-Gen, die zu einer Aminosäurensubstitution *p.(ASN188Asp) *führt*.* Sie wird in SIFT und POLYPHEN als pathogen angeführt und wurde bereits in der Literatur bei einem Patienten mit epidermolytischer Ichthyose berichtet [3].

## Wie lautet Ihre Diagnose?

**Diagnose:** Epidermolytische Ichthyose

## Diagnose

Somit konnte die klinische Verdachtsdiagnose einer epidermolytischen Ichthyose auf molekularer Ebene bestätigt werden.

## Therapie und Verlauf

Es erfolgte eine umfangreiche Aufklärung der Eltern über Prognose, Verlauf und Therapieoptionen sowie eine genetische Beratung. Der Kontakt mit der deutschen Selbsthilfegruppe Ichthyose e. V. und einer anderen betroffenen Familie in Österreich wurde vermittelt.

Während des 4‑wöchigen Aufenthaltes an der Intensivstation erhielt das Kind eine analgetische Therapie mit Morphin, eine antibiotische Therapie bei Erhöhung der Entzündungswerte, Elektrolyt‑, Vitamin- und Eisensubstitutionen sowie eine hochkalorische Ernährung. Die Lokaltherapie erfolgte mit einer Dexpanthenol-haltigen Wund- und Heilsalbe 5‑mal täglich.

**Figure 2:**
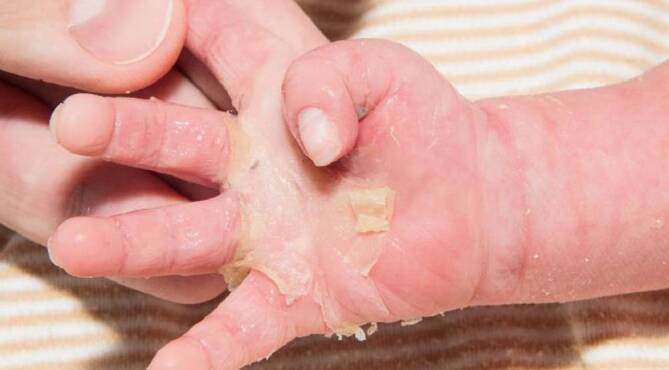

Bei einer Kontrolle 6 Wochen später zeigte der Säugling eine Erythrodermie mit vereinzelten Erosionen und palmoplantaren Hyperkeratosen (Abb. 2 und 3). Anamnestisch berichtete die Mutter über rezidivierende Blasenbildung. Die Lokaltherapie erfolgte mit Sonnenblumenöl in Unguentum Cordes, Ölbädern und antiseptischem Wundmanagement.

**Figure 3:**
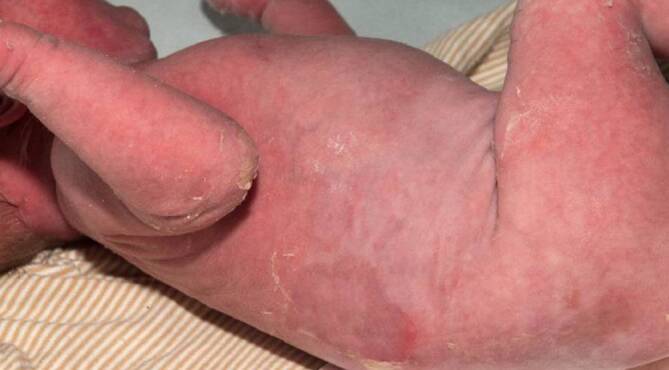

## Definition

Ichthyosen sind eine Gruppe genetisch bedingter Hauterkrankungen, denen eine Störung der Barrierefunktion und folgend eine pathologische Verhornung gemeinsam ist. Sie werden in nichtsyndromale und syndromale Ichthyosen eingeteilt, wobei Letztere mit einer Beteiligung anderer Organe einhergehen [1].

Die seltene epidermolytische Ichthyose zählt zu den nichtsyndromalen Ichthyosen und wird durch Mutationen in den Genen Keratin‑1 (*KRT1*) oder Keratin-10 (*KRT10*) verursacht. Sie wird überwiegend autosomal-dominant vererbt. Die epidermolytische Ichthyose manifestiert sich bei Geburt mit einer kongenitalen ichthyosiformen Erythrodermie mit einem unterschiedlichen Ausmaß an Blasen und Erosionen (Bild des „verbrühten Kindes“). Im Kindesalter nimmt die Blasenbildung ab, und es entwickelt sich eine zunehmende Hyperkeratose, v. a. im Nacken, um die großen Beugen und an den Extremitätenstreckseiten. Kinder mit *KRT1*-Muationen zeigen zudem eine diffuse palmoplantare Hyperkeratose [2].

## Fazit

Bei Neugeborenen mit Erythrodermie und Blasenbildung muss an eine epidermolytische Ichthyose gedacht werden. Es ist eine intensivmedizinische Überwachung wegen der erhöhten Infektionsgefahr durch die gestörte Hautbarrierefunktion und die ausgedehnten Erosionen notwendig [4].

Differenzialdiagnostisch stellen kutane Infektionen die häufigste Ursache für Erosionen und Blasen in der Neonatalperiode dar, weshalb eine ausführliche Erregerdiagnostik essenziell ist.

Eine schnelle Diagnose bei Neugeborenen mit Verdacht auf eine Genodermatose ist wichtig für eine frühe Information der Eltern über Prognose und Verlauf, gezielte humangenetische Beratung, Kostenersatz für Medikamente sowie die Kontaktaufnahme zu Selbsthilfegruppen und stellt eine Entscheidungshilfe für weitere Untersuchungen und zielgerichtete Therapien dar. Die molekulargenetische Analyse zur Diagnostik von Ichthyosen hat andere invasive Methoden in den Hintergrund gestellt. Der Nutzen von Hautbiopsien beim Neugeborenen muss aufgrund der erhöhten Infektionsgefahr streng abgewogen werden [4, 5].

Die Therapie der epidermolytischen Ichthyose erfolgt im Kindesalter mit intensiven lokaltherapeutischen Maßnahmen, orale Retinoide sind üblicherweise erst im Erwachsenenalter erforderlich [5]. Hoffnungsvolle zukünftige Behandlungsansätze sind Enzymersatz- und Gentherapien, diese befinden sich allerdings noch in einem frühen Entwicklungsstadium.
